# Blood‐based plasmatic aminoacid profiles as potential biomarkers for dementia

**DOI:** 10.1002/alz.093365

**Published:** 2025-01-09

**Authors:** André Luiz Rodrigues Palmeira, Liana Lisboa Fernandez, Mayra Pacheco Pachado, Leonardo Martins de Paula, Carolina Fontela, Tiago Gil Oliveira, Sarah Eller

**Affiliations:** ^1^ Universidade Federal de Ciências da Saúde de Porto Alegre, Porto Alegre, RS Brazil; ^2^ Universidade Federal de Ciências da Saúde de Porto Alegre, Porto Alegre, Rio Grande do Sul Brazil; ^3^ Maastricht University, Maastricht, Limburg Netherlands; ^4^ Hospital de Clínicas de Porto Alegre, Porto Alegre, Rio Grande do Sul Brazil; ^5^ Universidade Federal de Ciências da Saúde de Porto Alegre, porto Alegre, RS Brazil; ^6^ School of Medicine, Institute of Life and Health Sciences (ICVS), University of Minho, Braga Portugal

## Abstract

**Background:**

The study of new blood biomarkers in addition to amyloid beta and hyperphosphorylated tau is fundamental to expanding the understanding of the pathophysiology of dementia, especially Alzheimer`s disease, in search of therapeutic approaches.

**Method:**

In this study, we included individuals diagnosed with Alzheimer disease (AD), Mixed‐type dementia (MD), Vascular dementia (VD) and a control group of cognitively healthy individuals. Demographics, clinical characteristics, neuroimages and plasma samples were collected. We performed a metabolomic aminoacid analysis using High‐Performance Liquid Chromatography‐Mass Spectrometry (HPLC‐MS/MS). Analyses were performed with GraphPad Prism 9.0 (GraphPad Software, San Diego, USA) or SPSS v. 18 software. Depending on the group comparison type, one‐way analysis of variances with post‐hoc Tukey‐HSD test or independent samples t‐tests. We compared aminoacid levels between the different groups (controls, AD, MD or VD) and dementia severity, assessed by Clinical Dementia Rating (CDR) score.

**Result:**

135 individuals were clinically assessed as 33 controls, 42 AD, 41 MD and 19 VD. There was a statistically significant difference between controls and individuals with dementia in the following amino acids: aspartic acid (*p<0.0001*), arginine (*p<0.0001*), glutamic acid (*p<0.0001*), glutamine (*p<0.0001*), isoleucine (*p<0.0008*), leucine (*p<0.0001*). However, only arginine proved to be a biomarker capable of differentiating AD from DV (*p<0.0003*)

**Conclusion:**

These preliminary analyses show that leucine, isoleucine and glutamine were reduced in the plasma of patients with dementia, while aspartic acid, glutamic acid and arginine were increased. These markers can help us identify the underlying metabolic dysfunctions of different signaling pathways relevant to neuroprotection and vascular function.

